# The DNA repair function of *CUX1* contributes to radioresistance

**DOI:** 10.18632/oncotarget.14875

**Published:** 2017-01-28

**Authors:** Zubaidah M. Ramdzan, Vasudeva Ginjala, Jordan B. Pinder, Dudley Chung, Caroline M. Donovan, Simran Kaur, Lam Leduy, Graham Dellaire, Shridar Ganesan, Alain Nepveu

**Affiliations:** ^1^ Goodman Cancer Research Centre, McGill University, Montreal, Quebec, H3A 1A3, Canada; ^2^ Department of Biochemistry, McGill University, Montreal, Quebec, H3A 1A3, Canada; ^3^ Department of Medicine, McGill University, Montreal, Quebec, H3A 1A3, Canada; ^4^ Department of Oncology, McGill University, Montreal, Quebec, H3A 1A3, Canada

**Keywords:** CUX1, OGG1, base excision repair, radioresistance

## Abstract

Ionizing radiation generates a broad spectrum of oxidative DNA lesions, including oxidized base products, abasic sites, single-strand breaks and double-strand breaks. The CUX1 protein was recently shown to function as an auxiliary factor that stimulates enzymatic activities of OGG1 through its CUT domains. In the present study, we investigated the requirement for CUX1 and OGG1 in the resistance to radiation. Cancer cell survival following ionizing radiation is reduced by *CUX1* knockdown and increased by higher *CUX1* expression. However, *CUX1* knockdown is sufficient by itself to reduce viability in many cancer cell lines that exhibit high levels of reactive oxygen species (ROS). Consequently, clonogenic results expressed relative to that of non-irradiated cells indicate that *CUX1* knockdown confers no or modest radiosensitivity to cancer cells with high ROS. A recombinant protein containing only two CUT domains is sufficient for rapid recruitment to DNA damage, acceleration of DNA repair and increased survival following radiation. In agreement with these findings, OGG1 knockdown and treatment of cells with OGG1 inhibitors sensitize cancer cells to radiation. Together, these results validate CUX1 and more specifically the CUT domains as therapeutic targets.

## INTRODUCTION

Approximately half of all cancer patients receive ionizing radiation as a part of treatment. Radiotherapy remains the most effective nonsurgical treatment for most solid tumors [1]. Repair of DNA lesions caused by radiation involves several DNA repair pathways including base excision repair (BER), classical and alternate non-homologous end-joining (NHEJ) and homology-dependent repair (HDR). Radiation causes DNA damage through direct ionization of DNA and indirectly, through ionization of water to produce hydroxyl radicals [2]. Early studies using ·OH radical scavengers established that 65% of cell killing resulting from low linear energy transfer (LET) radiation, such as X-rays or γ-rays, was caused by hydroxyl radicals [3, 4]. Ionizing radiation tends to create clusters of DNA damage including single-strand breaks, abasic sites or oxidized purines and pyrimidines within one or two helical turns of the DNA [5–7]. Double-strand breaks (DSBs), which are believed to be the predominant cytotoxic lesions, are produced when two single-strand breaks (SSBs) are close to each other on alternate DNA stands. Analysis of radiation-induced DNA damage using lesion-specific enzymes and pulse-field gel electrophoresis established that double-strand breaks represent only about 20% of clustered damage sites, with the remaining 80% being non-DSB clusters ([8–11], reviewed in [12, 13]). Replication through a SSB generates one-ended DSB, described as a “double-strand end” [14]. Moreover, repair of clustered oxidized bases or abasic sites by base excision repair (BER) can also produce secondary DSBs [15]. There is much evidence to show that clustered DNA lesions are more difficult to repair and therefore persist longer [16–20], reviewed in [12, 13]. This is in stark contrast to the efficient repair of isolated DNA lesions produced by endogenous reactive oxygen species [21]. The reduced repairability and the heightened lifetime of DNA lesions within clusters increase the probability of an encounter with a replication fork which would result in the generation of a double-strand end [22, 23]. In addition to radiation-induced cytotoxicity resulting from DSBs and non-DSB clusters, replicating blocking lesions such as thymine glycol, 4, 6-diamino-5-formamidopyrimidine (FapyA) and 2, 6-diamino-4-hydroxy-5-formamidopyrimidine (FapyG), are toxic and can be lethal if not rapidly repaired [24–27].

BER is the major pathway responsible for the repair of isolated base damage and non-DSB clustered DNA damage sites [28]. This pathway is initiated by one of many DNA glycosylases that recognize specific types of altered bases and cleave the N-glycosylic bond linking the altered base to the DNA backbone [29, 30]. These reactions produce AP sites which are targeted by the AP endonuclease 1, APE1, which incises the DNA backbone immediately 5′ to the AP site, generating a 5′-deoxyribose-5-phosphate (5′-dRP) product that will be processed by DNA Polβ [31, 32]. Oxidative purine lesions are removed primarily by OGG1, whereas oxidative pyrimidine lesions are removed primarily by NTH1, NEIL1, or NEIL2 (reviewed in [29, 33, 34]). Although each of these glycosylases exhibits substrate preference, none has absolute specificity (reviewed in [35]). DNA glycosylases for oxidized bases are bifunctional, and are endowed with both a glycosylase and an AP/lyase activity that generates a single-strand nick 3′ to the AP site via beta (OGG1, NTH1) or beta-delta (NEIL1, NEIL2) elimination. 5′ or 3′ end-processing of the resulting single-strand breaks are then performed by PNKP or APE1, respectively [36–38]. The gap is filled in by a DNA polymerase and sealed by a DNA ligase [34, 39].

The Cut homeobox 1 (*CUX1*) gene has been characterized as a haploinsufficient tumor suppressor gene, but is also overexpressed in many advanced cancers (reviewed in [40]). The dual role of CUX1 in cancer is illustrated by the fact that many cell lines with loss-of-heterozygosity of *CUX1* display amplification of the remaining allele, suggesting that decreased *CUX1* expression facilitates tumor development while increased *CUX1* expression promotes cancer cell survival and tumor progression. The molecular functions of CUX1 that explain its dual role in cancer remain to be clarified. *CUX1* codes for an abundant protein, often called p200 CUX1, and several much less abundant protein isoforms, collectively called p110 CUX1, that are generated by proteolytic processing [41, 42]. Shorter CUX1 protein isoforms have been characterized as transcription factors that bind stably to DNA and function as activators or repressors depending on promoter context [43, 44]. Transcription and cell-based assays established a role for p110 CUX1 in many cellular processes, including cell cycle progression and cell proliferation [45, 46], strengthening of the spindle assembly checkpoint [47], establishment of a transcriptional program that enables efficient DNA damage responses [48], and cell migration and invasion [49, 50].

The full-length protein, p200 CUX1, contains four evolutionarily conserved DNA binding domains: three CUT domains, C1, C2 and C3 (also called Cut repeats) and a Cut homeodomain (HD) [51]. p200 CUX1 is very abundant and binds DNA with extremely fast kinetics [52]. These properties are not consistent with a role as a classical transcription factor. We have established that p200 CUX1 plays a direct role in DNA repair through its three CUT domains. CUT domains were shown to stimulate the glycosylase and AP/lyase activities of OGG1 [53–55]. The importance of CUX1 in the repair of oxidative DNA damage is illustrated by the fact that mouse embryo fibroblasts from Cux1^−/−^ knockout mice senesce immediately when placed in 20% oxygen, although they proliferate very well in 3% oxygen [55]. On the other hand, higher *CUX1* expression in RAS-driven cancer cells that produce elevated levels of reactive oxygen species enables rapid repair of oxidative DNA damage, thereby preventing cellular senescence and allowing proliferation [53].

In the present study, we investigated the role of *CUX1*, in particular its DNA repair function, in the resistance of cancer cells to ionizing radiation. We found that *CUX1* knockdown sensitizes cancer cells to radiation, whereas overexpression confers resistance. To investigate the contribution of its DNA repair function, we ectopically expressed a recombinant protein containing only two CUT domains, C1C2, that had previously been shown to be devoid of transcriptional potential [53, 55]. The C1C2 protein was rapidly recruited to the site of DNA damage and in DLD-1 colorectal cells, stimulated OGG1 activity and increased resistance to radiation. Previous studies showed that ectopic expression of OGG1 and NTH1 sensitizes TK6 cells to radiation [56–58]. However, we found that OGG1 overexpression protects against radiation in DLD-1 cells, which express high levels of enzymes involved in downstream steps of base excision repair. We propose that the opposite effect of OGG1 overexpression in different cell lines is due to the fact that some cancer cells adapt to high levels of reactive oxygen species by enhancing BER activity. Importantly, OGG1 knockdown or inhibition, like *CUX1* knockdown, sensitized DLD-1 cancer cells to radiation.

## RESULTS

### *CUX1* knockdown further reduces tumor cell survival following ionizing radiation

**Figure 1:**
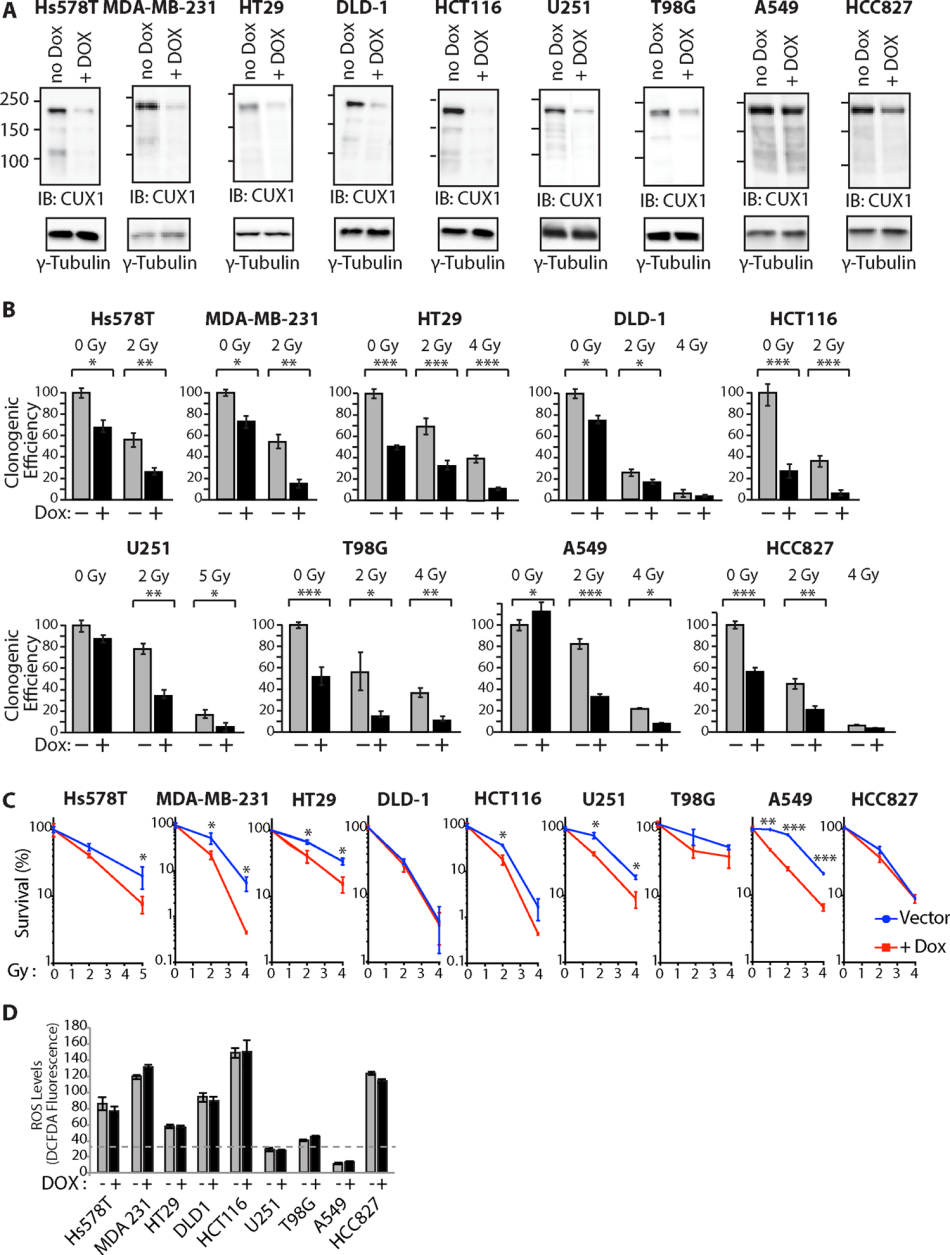
CUX1 Knockdown Sensitizes Tumor Cells to Radiation Lentivirus expressing a doxycycline inducible shRNA against CUX1 was introduced in tumor cell lines of various tissue of origin to obtain large populations of cells stably carrying the lentiviral vector. Cells were treated with doxycycline (+) or not (−) for 4 days. (**A**) Total protein extracts were used in immunoblotting analysis using the indicated antibodies. (**B**) Cells were treated with radiation and then submitted to a clonogenic assay. Cloning efficiency of untreated control cells was set at 100%. Results of triplicate experiments are shown. Error bars represent standard error. ***p < 0.001; **p < 0.01.; *p < 0.05; Student's *t*-test. (**C**) Results from clonogenic efficiency data in Figure 1B are represented as line graphs with all un-irradiated cells (−Dox, +Dox) set at 100%. Results of triplicate experiments are shown. Error bars represent standard error. ***p < 0.001; **p < 0.01.; *p < 0.05; Student's *t*-test. (**D**) Levels of reactive oxygen species (ROS) were measured by staining with CM-H_2_DCFDA.

To investigate the requirement for *CUX1* in the resistance to radiation, we established populations of tumor cell lines stably carrying a lentiviral vector expressing a *CUX1* shRNA under the control of a doxycycline-inducible promoter. CUX1 protein expression was reduced upon treatment with doxycycline (Figure 1A). Upon irradiation, *CUX1* knockdown reduced clonogenic efficiency in all tested tumor cell lines (Figure 1B).

Closer examination of the results, however, revealed a more complex situation since CUX1 knockdown also reduced clonogenic efficiency in the absence of radiation in 7 out of 9 tumor cell lines: Hs578T, MDA-MB-231, HT29, DLD-1, HCT116, T98G and HCC827 (Figure 1B). To take this effect into consideration, the impact of CUX1 knockdown on survival after irradiation was also expressed relative to that of non-irradiated cells (Figure 1C). The results show that *CUX1* knockdown does not affect radiosensitivity in DLD-1, T98G and HCC827 cells, while it has a modest effect in MDA-MB-231, HT29, U251 and A549 cells (Figure 1C).

These observations led us to seek the molecular basis for the synthetic lethality of CUX1 knockdown in a subset of cancer cell lines. A previous study showed that the synthetic lethality of CUX1 knockdown in RAS-driven cancer cells was associated with an increase in oxidative DNA damage caused by high levels of reactive-oxygen species (ROS) [55]. High ROS levels were also observed in many of the cell lines that carry an oncogene that activates the RAS pathway: Hs578T^HRAS^, MDA-MB-231^HRAS^, HT29^BRAF^, DLD-1^KRAS^, HCT116^KRAS^ and HCC827^EGFR^ (Figure 1D). Importantly, CUX1 knockdown did not decrease ROS levels (Figure 1D). These results suggest that the same mechanism, an excess of oxidative DNA damage, explains the decrease in clonogenic efficiency observed in these cell lines following CUX1 knockdown. However, we note that while clonogenic efficiency is decreased by *CUX1* knockdown in all cell lines that exhibit ROS levels over a certain threshold, we do not observe a direct correlation between ROS levels and the effect of *CUX1* knockdown. This suggests that while CUX1 is needed for optimal proliferation in cells with elevated ROS, other proteins must play an important role in protecting cells against deleterious effects of ROS. The synthetic lethality of CUX1 knockdown in T98G cells probably involves a different mechanism that will be addressed in the discussion.

### Ectopic expression of p200 CUX1 increases tumor cell survival following ionizing radiation

**Figure 2:**
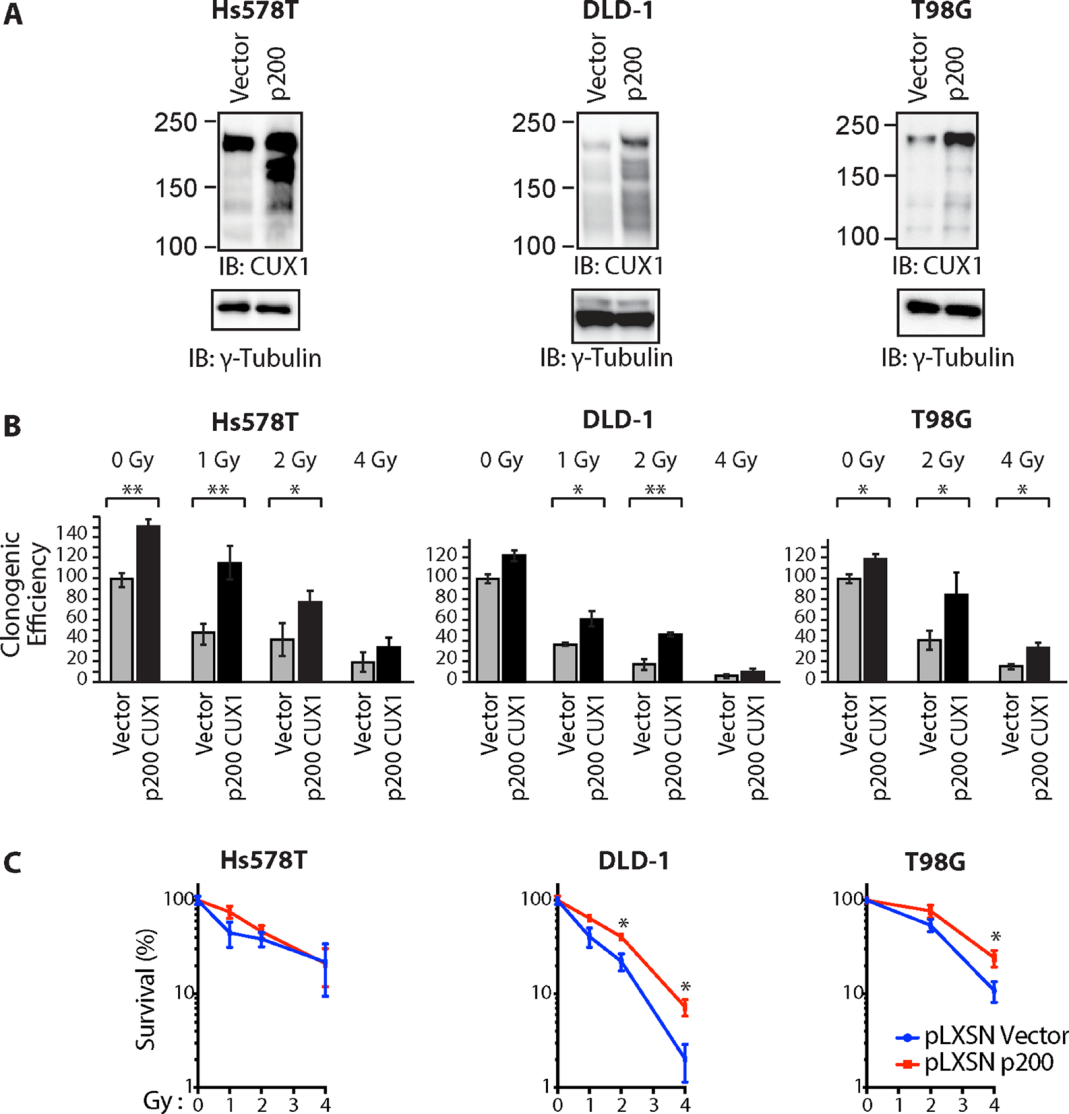
Ectopic expression of p200 CUX1 confers resistance to radiation Tumor cells were stably infected with retroviruses expressing p200 CUX1-HA or nothing (vector). (**A**) Expression of recombinant CUX1 protein expression was analyzed by immunoblotting using CUX1 (861) antibodies. (**B**) Cells were treated with radiation and then submitted to a clonogenic assay. Results of triplicate experiments are shown. Error bars represent standard error. ***p < 0.001; **p < 0.01.; *p < 0.05; Student's *t*-test. (**C**) Cloning data in Figure 2B is represented as line graphs where both untreated cells (control cells and cells overexpressing p200) were set at 100%. Results of triplicate experiments are shown. Error bars represent standard error. ***p < 0.001; **p < 0.01.; *p < 0.05; Student's *t*-test.

To investigate whether higher CUX1 expression would increase resistance to radiation, we established populations of Hs578T, DLD-1 and T98G tumor cells stably carrying either a retroviral vector expressing p200 CUX1 or an empty vector (Figure 2A). Ectopic expression of p200 CUX1 increased clonogenic efficiency following irradiation in the three tested tumor cell lines (Figure 2B). However, as p200 CUX1 increased clonogenic efficiency of non-irradiated cells, the results were also expressed relative to that of non-irradiated cells (Figure 2C).

### p200 CUX1 is rapidly recruited to DNA damage sites

To investigate whether p200 CUX1 plays a direct role in the response to DNA damage, we expressed a fusion protein containing the green fluorescent protein fused to the C-terminus of p200 CUX1 and submitted cells to laser-microirradiation at 351/364 nm and 405 nm. We reasoned that both types of radiation would cause many types of DNA damage, including double-strand breaks (DSBs), single-strand breaks (SSBs), abasic sites, oxidized purines and pyrimidines and their derivatives. Our rationale in choosing these conditions was to reproduce the effects imparted on cells by the radiation treatment employed in the clonogenic assay. Of relevance to the present study, recruitment of OGG1 to DNA lesions has previously been documented following laser micro-irradiation with similar conditions [59–61].

**Figure 3:**
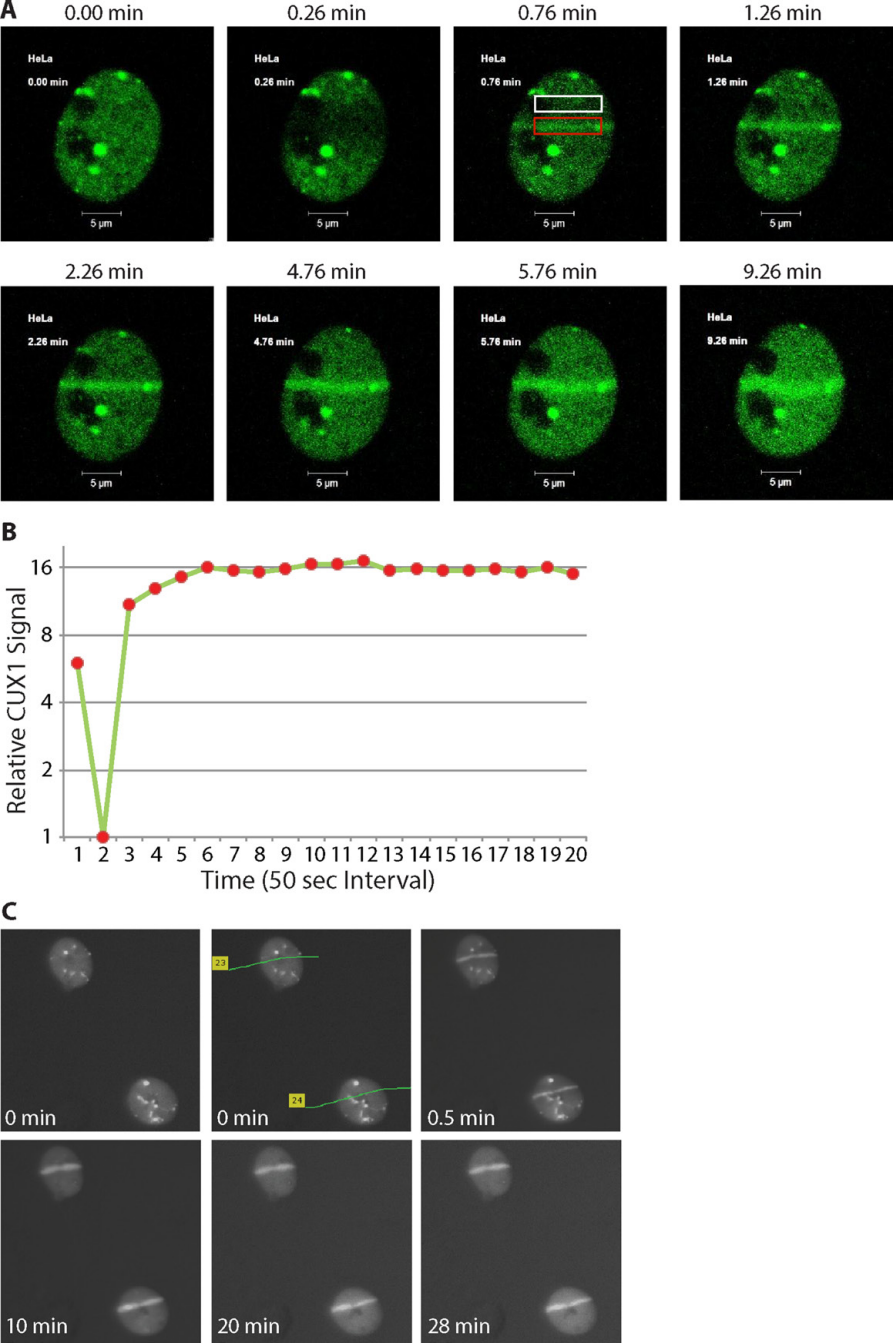
p200 CUX1 is rapidly recruited to DNA damage sites (**A**) Cells were transfected with a plasmid expressing a p200 CUX1-GFP fusion protein. DNA was damaged using 351/364 nm laser micro-irradiation and images were acquired immediately before DNA damage and periodically thereafter using the Argon laser (488 nm). See Supplementaru Movie 1. (**B**) The pixel intensity in the red rectangular box in Figure 3A surrounding the DNA damage was measured using Adobe Photoshop CS6. Normalized pixel intensity in region of damage was compared to undamaged region (Figure 3A; white box), called the relative CUX1-GFP signal was plotted as a function of time. (**C**) Cells were submitted to 337 nm laser micro-irradiation and were either fixed immediately or returned to the incubator and fixed at the desired time point followed by immunocytochemical staining.

Cells were submitted to 351/364 nm laser micro-irradiation to induce focal regions of DNA damage, and images were taken at different time before and after irradiation. GFP-p200 CUX1 images show that while the protein was distributed throughout the nucleus prior to irradiation (Figure 3A, 0.00 panel), it became associated with DNA damage in less than a minute and persisted there until the last image was taken at 9.26 min. (Figure 3A and 3B; see Supplementary Movie 1). We repeated the experiment, this time using 337 nm laser and immunocytochemistry as a method of detection. CUX1 proteins were recruited to the focal region of DNA damage in less than a minute and persisted on DNA damage for at least 28 minutes (Figure 3C). Together, these results show that CUX1 proteins are rapidly recruited to the sites of DNA damage.

### Cut domains are sufficient for the recruitment to DNA damage sites

**Figure 4:**
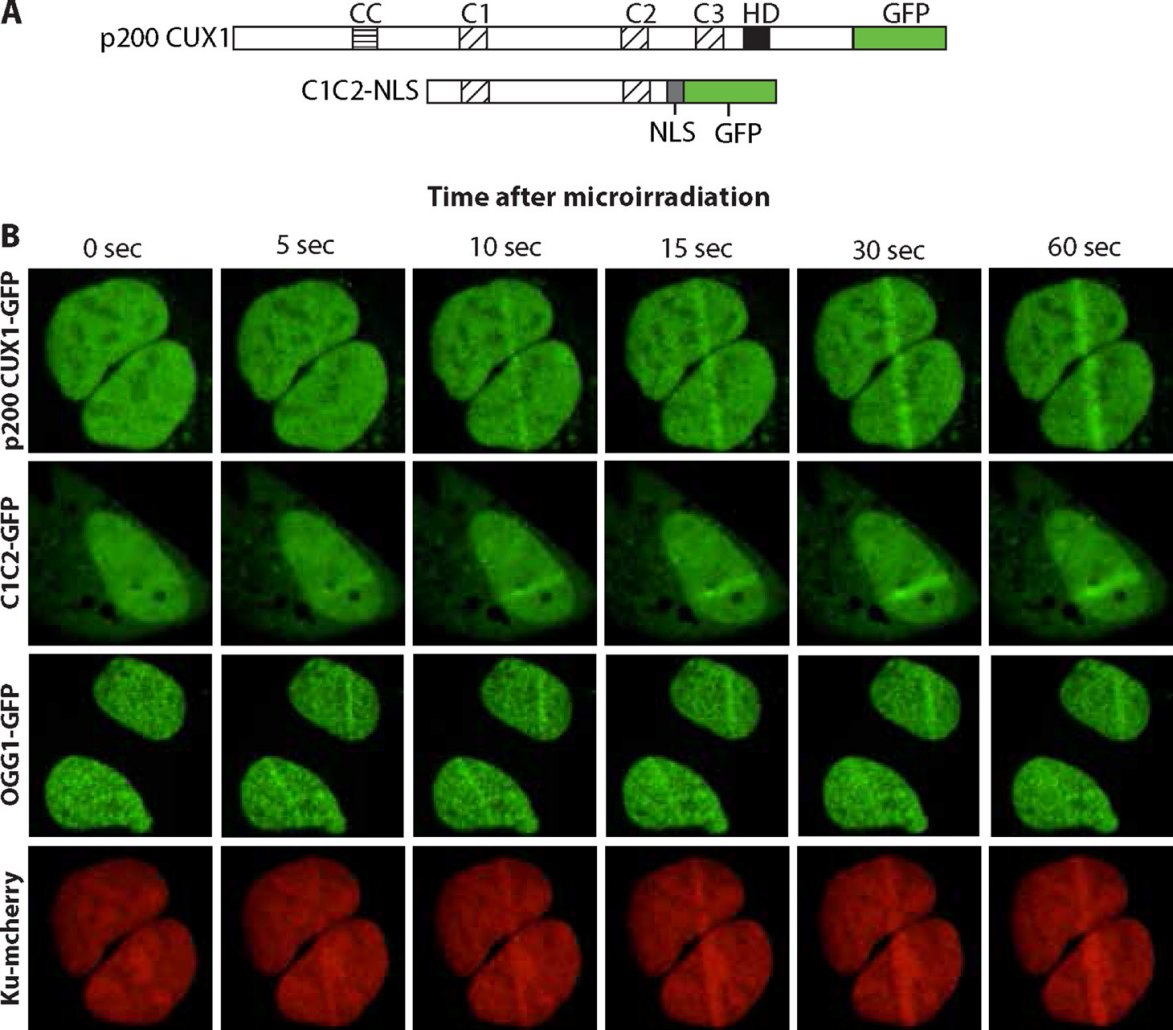
CUT domains are sufficient for the recruitment to DNA damage sites (**A**) Diagrammatic representation of the constructs. The evolutionarily conserved domains of CUX1 are shown: CC, coiled-coil; C1, C2 and C3, CUT domains 1, 2 and 3; HD, homeodomain. NLS, nuclear localization signal. In the absence of the Cut homeodomain, a nuclear localization signal (NLS) is required to target C1C2 to the nucleus. (**B**) U2OS cells were transfected with plasmids expressing p200 CUX1-GFP, C1C2-NLS-GFP, OGG1-GFP, or Ku-mcherry fusion proteins. A 405 nm UV laser was used to induce DNA damage and the recruitment of GFP fusion proteins to the site of damage was followed in real-time. See Supplementary Movies 2–5.

To confirm recruitment of CUX1 to DNA damage, we performed similar assays using 405 nm laser irradiation. In parallel, we also expressed another GFP fusion protein that only contained CUT domains 1 and 2 of CUX1, C1C2. A nuclear localization signal (NLS) was added to enable nuclear localization in the absence of the Cut homeodomain (Figure 4A). Both p200 CUX1-GFP and C1C2-GFP proteins were rapidly recruited to the focal regions of induced DNA damage (Figure 4B; see Supplementary Movies 2 and 3). These results established that CUT domains are sufficient for rapid recruitment to DNA damage. Importantly, similar experiments using cells expressing OGG1-GFP and Ku-mcherry show that the two proteins were recruited to the micro-irradiated region, thereby establishing that both double-strand break and oxidized base DNA lesions were produced using these micro-irradiation conditions (Figure 4B; see Supplementary Movies 4 and 5).

### Cut domains and OGG1 accelerate repair of oxidative DNA damage and increase tumor cell survival following ionizing radiation

**Figure 5:**
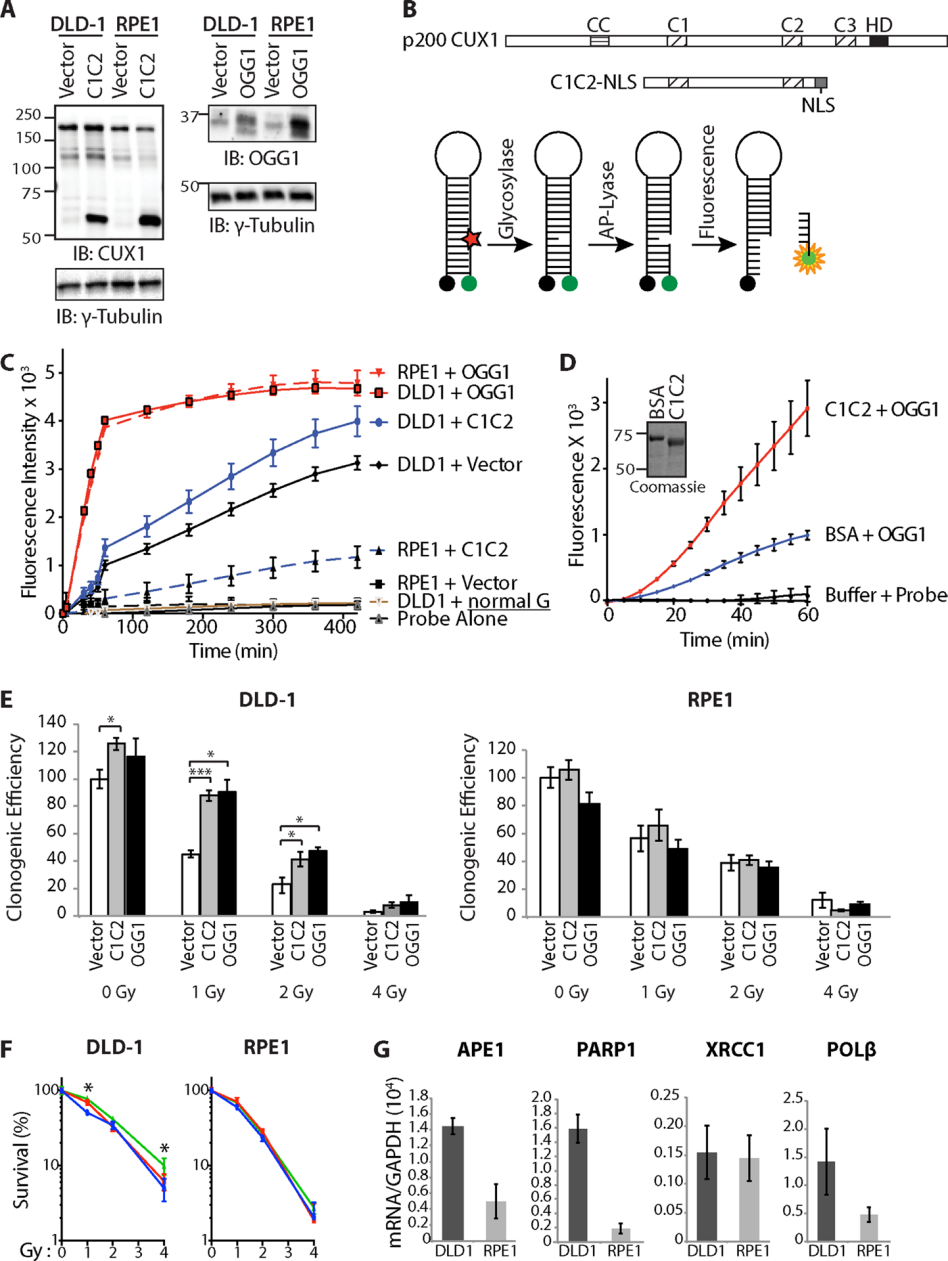
Cut domains and OGG1 Accelerate Repair of Oxidative DNA Damage and Increase Resistance to Radiation (**A**) DLD-1 (KRAS^G13D^) and RPE1 cells were infected with retroviruses expressing either nothing (Vector), C1C2-NLS or OGG1. Nuclear extracts were analyzed by immunoblotting using the indicated antibodies. (**B**) Map showing the evolutionarily conserved domains of CUX1:CC, coiled-coil; C1, C2, C3, CUT domain 1, 2 and 3; HD, homeodomain. The CUT domain 1 and 2 (C1C2) construct includes a nuclear localization signal (NLS). Diagrammatic representation of the 8-oxoG cleavage assay using a fluorophore reporter probe. Red star, 8-oxoguanine; green circle, FAM fluorophore; black circle, dabcyl quencher. (**C**) 8-oxoG cleavage assay was performed using whole cell extracts from DLD-1 and RPE1 cells stably carrying vectors expressing either OGG1, CUT domains 1 and 2 (C1C2) or nothing (vector). Two controls are shown. “Probe Alone” is the probe incubated in the absence of cell extract. “DLD1+normal G” is a reaction with DLD1 cell extract and a probe that contains a normal guanine residue instead of 8-oxoG; the lack of fluorescence confirms that that the increase in fluorescence intensity in the reactions with the 8-oxoG probe is not caused by a nonspecific nuclease activity. (**D**) 8-oxoG cleavage assay was performed using 50 nM OGG1 and 200 nM of BSA or recombinant CUT domains 1 and 2 (C1C2). A coomassie blue stain of the C1C2 purified protein is shown. (**E**) Cells were exposed to radiation and then submitted to a clonogenic assay. Cloning efficiency of unexposed cells was set at 100%. Results of triplicate experiments are shown. Error bars represent standard error. ***p < 0.001; **p < 0.01.; *p < 0.05; Student's *t*-test. (**F**) Cloning data in Figure 5E is represented as line graphs where non-irradiated cells were set at 100%: control cells (blue), cells overexpressing C1C2 (red) or OGG1 (green). Results of triplicate experiments are shown. Error bars represent standard error. ***p < 0.001; **p < 0.01.; *p < 0.05; Student's *t*-test. (**G**) RT-PCR analysis was performed to measure mRNA levels of genes involved in base excision repair. All mRNA levels were normalized to glyceraldehyde 3-phosphate dehydrogenase (GAPDH). The values are the mean of three measurements and error bars represent standard deviation.

Tumor cell survival following ionizing radiation was shown to decrease following CUX1 knockdown but to increase following ectopic expression of the full-length p200 CUX1 protein (Figures 1 and 2), while results from laser micro-irradiation indicate that CUX1 proteins are recruited to DNA damage. Together these results suggest that the direct role of CUX1 proteins in DNA repair may influence the extent to which cancer cells exhibit resistance to radiation. However, up to 5% of CUX1 can be proteolytically processed to generate the p110 CUX1 isoform, which transcriptionally activates many genes involved in the DNA damage response [48]. Therefore, in addition to its direct role in DNA repair, CUX1 also regulates a transcriptional program that is necessary to mount an efficient response to DNA damage [48]. To investigate the impact of CUX1 DNA repair function on radioresistance, we established populations of DLD-1 colorectal cancer cells and retinal pigment epithelial (RPE1) cells stably expressing the CUT domains 1 and 2 protein, C1C2-NLS (Figure 5A and 5B). The C1C2-NLS protein was previously shown to be devoid of transcriptional activation potential, both in DLD-1 cells and in mouse embryo fibroblasts [53, 55]. In parallel, we also established populations of cells stably expressing human OGG1 (Figure 5A).

Whole cell extracts from each cell population were used in 8-oxoG cleavage assay using a fluorophore-based probe (Figure 5B). Ectopic expression of either OGG1 or the recombinant C1C2 CUX1 protein increased the efficiency of 8-oxoG cleavage both in DLD-1 and RPE1 cells (Figure 5C). The same assay performed with purified proteins demonstrated that CUT domains 1 and 2 stimulate the enzymatic activities of OGG1 (Figure 5D).

Ectopic expression of either OGG1 or the recombinant C1C2 CUX1 protein increased survival following radiation in DLD-1 cells, but not in RPE1 cells (Figure 5E). Since C1C2 CUX1 and OGG1 increased clonogenic efficiency of untreated cells, survival after irradiation was also represented as a percentage to that of non-irradiated cells (Figure 5F). Analysis of gene expression by reverse-transcriptase quantitative PCR analysis showed that DLD-1 cells express higher levels of many genes involved in downstream steps of base excision repair, including APE1, PARP1 and DNA pol β (Figure 5G). These findings likely explain that OGG1 overexpression increased survival after radiation in DLD-1 cells but not in RPE1 cells.

### A CUT domain mutant that is impaired in its ability to stimulate OGG1 is also less able to increase survival after ionizing radiation

**Figure 6:**
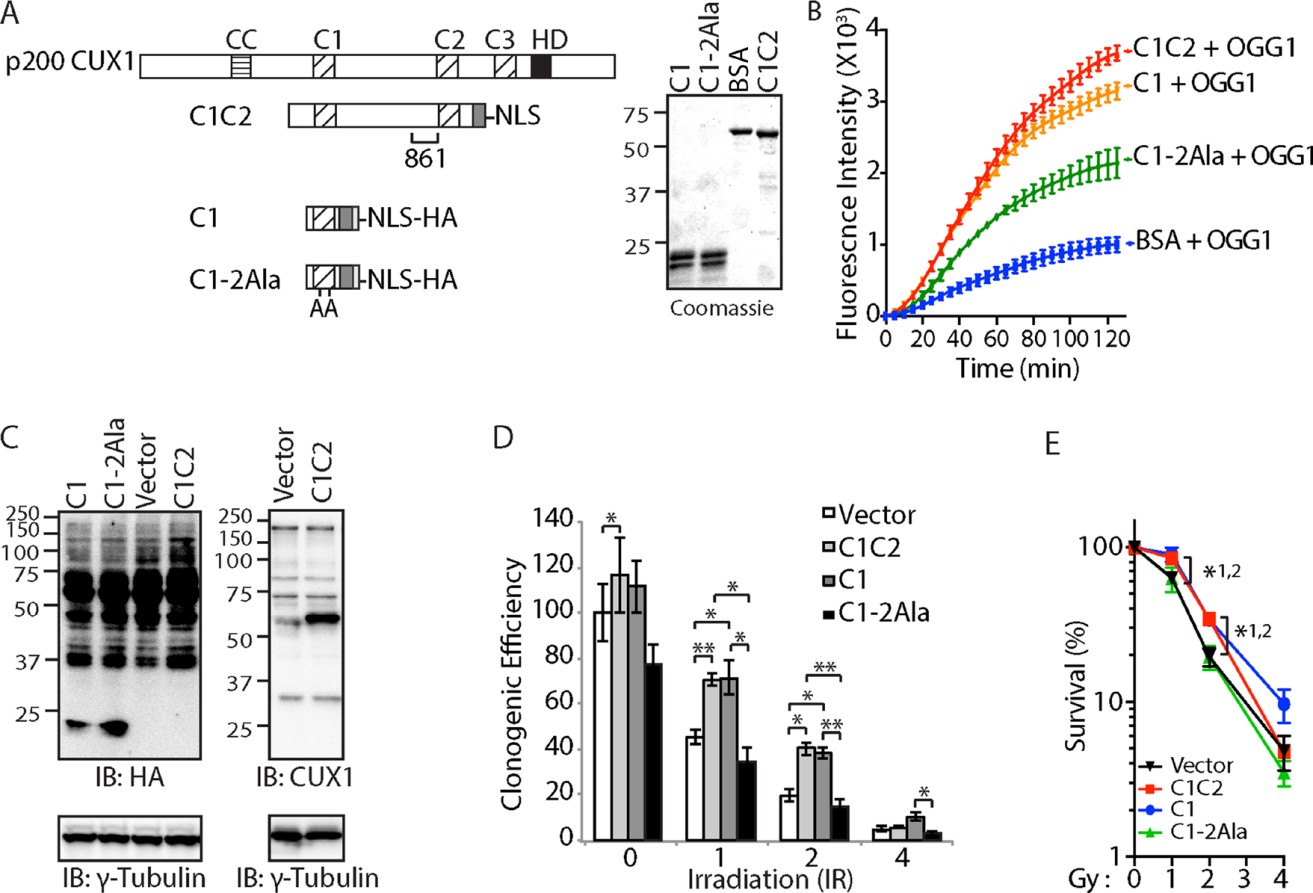
Cut domain is Necessary to Increase Resistance to Radiation and Repair of Oxidative DNA Damage (**A**) Map showing the recombinant CUX1 proteins C1C2, C1 and C1-2Ala, which contains two point mutations replacing glutamic acid to alanine. The region recognized by the CUX1-861 antibody is shown below the C1C2 map. A coomassie blue stain of the purified proteins is shown. (**B**) 8-oxoG cleavage assay was performed using 50 nM OGG1 and 100 nM of BSA or C1C2, C1, or C1-2Ala. (**C**) DLD-1 cells were infected with lentiviruses expressing either nothing (Vector), C1C2-NLS, C1-NLS, or C1-2Ala-NLS. Nuclear extracts were analyzed by immunoblotting using the indicated antibodies. (**D**) Cells were submitted to ionizing radiation and their clonogenic efficiency was determined. Cloning efficiency of unexposed vector cells was set at 100%. Results of triplicate experiments are shown. Error bars represent standard error. ***p < 0.001; **p < 0.01.; *p < 0.05; Student's *t*-test. (**E**) Results from clonogenic efficiency data in Figure 6D are represented as line graphs, with all non-irradiated cells set at 100%. Results of triplicate experiments are shown. Error bars represent standard error. *p < 0.05; Student's *t*-test comparing C1C2-NLS (1), C1-NLS (2), or C1-2Ala-NLS against the vector.

To establish a correlation between OGG1 stimulation and increased survival after ionizing radiation, we sought to test the effect of a CUT domain mutant that does not stimulate OGG1 to the same extent. In a previous study of the CUX2 protein, we showed that the ability of a CUT domain to stimulate OGG1 was reduced following the introduction of two point mutations replacing glutamic acids to alanines [54]. In the present study, we have engineered the same point mutations with the CUT domain 1 of CUX1 (Figure 6A: C1-2Ala). A DNA repair assay with purified proteins and a probe containing an 8-oxoG confirmed that the replacement mutations reduced the ability of the C1 domain to stimulate OGG1 (Figure 6B). For expression in mammalian cells, a nuclear localization signal and a hematoglutinin (HA) tag were added to each coding sequence (Figure 6A). Immunoblotting analysis established that the C1-2Ala protein was expressed as well as the wild type C1 in DLD-1 cells (Figure 6C). Unfortunately, the C1C2 protein was masked by a cross-reacting protein in the HA-blot, but was clearly visible in an immunoblot performed with the CUX1-861 antibody (Figure 6C). Following ionizing radiation, cells expressing the wild type C1 and C1C2 recombinant proteins exhibited increased clonogenic efficiency (Figure 6D and 6E). In contrast, the clonogenic efficiency of cells expressing C1-2Ala was essentially equivalent to that of cells carrying the empty vector (Figure 6D and 6E). These results suggest that the ability to increase cancer cell survival following ionizing radiation is associated with the ability to stimulate OGG1.

### OGG1 knockdown or inhibition sensitizes cancer cells to radiation

**Figure 7:**
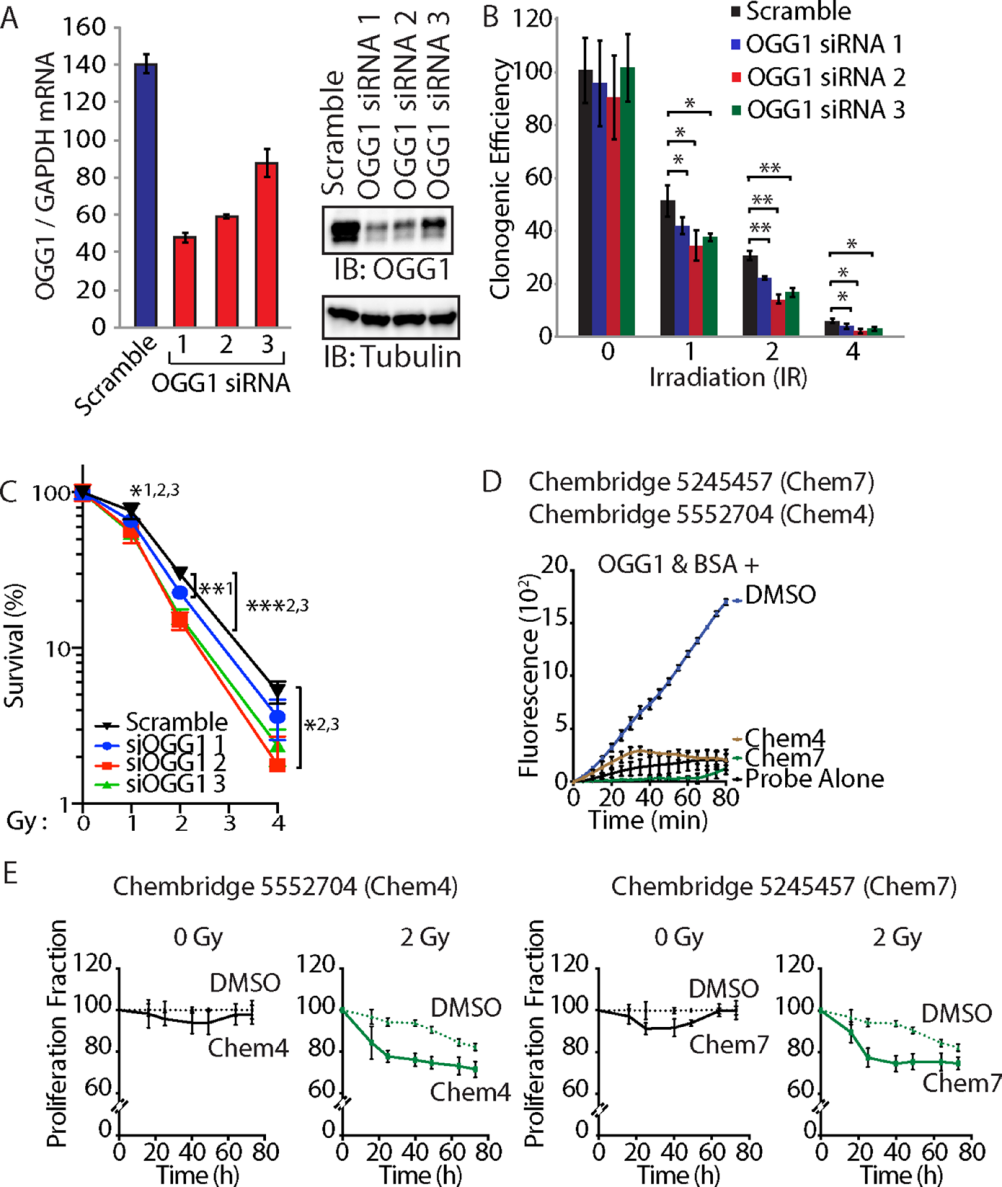
OGG1 knockdown or inhibition sensitizes cancer cells to radiation (**A**) DLD-1 cells were transfected with three distinct siRNA against OGG1 or with a scramble RNA. OGG1 expression was monitored by RT-qPCR analysis and immunoblotting. (**B**) Cells were submitted to irradiation and their clonogenic efficiency was determined. Cloning efficiency of unexposed cells was set at 100%. Results of triplicate experiments are shown. Error bars represent standard error. ***p < 0.001; **p < 0.01.; *p < 0.05; Student's *t*-test. (**C**) Results from clonogenic efficiency data in Figure 7B are represented as line graphs, with all un-irradiated cells set at 100%. Results of triplicate experiments are shown. Error bars represent standard error. ***p < 0.001; **p < 0.01.; *p < 0.05; Student's *t*-test comparing each siRNA OGG1 against the scramble RNA control. 1: OGG1 siRNA 1; 2 : OGG1 siRNA 2; 3: OGG1 siRNA 3. (**D**) 8-oxoG cleavage assay was performed using 50 nM OGG1 and BSA in the presence of 10 μM. Chembridge 5552704 and 5245457 compounds or vehicle (DMSO). The two compounds inhibit the reaction. (**E**) DLD-1 colorectal cancer cells were irradiated (0 and 2 Gy) in the presence of 10 μM of Chembridge 5552704 and 5245457 compounds or vehicle (DMSO). Thymidine incorporation was measured for 3 days and is reported relative to the DMSO 0 h value.

To investigate whether OGG1 activity is required for resistance to radiation, DLD-1 cancer cells were transfected with three distinct siRNAs that reduced OGG1 mRNA and protein expression (Figure 7A). Treatment with each of the three OGG1 siRNAs reduced clonogenic efficiency of DLD-1 cancer cells following radiation (Figure 7B and 7C). As an alternative approach to investigate the requirement for OGG1 in radioresistance, we tested two small molecules that were previously found to inhibit OGG1 enzymatic activities *in vitro* ([62] and Ramdzan and Nepveu, manuscript in preparation). As observed in the 8-oxoG cleavage assay using a fluorophore-based probe, both Chembridge 5245457 and 5552704 compounds inhibit OGG1 activity (Figure 7D). Treatment with Chembridge 5245457 and 5552704 compounds decreased the proliferation potential of DLD-1 cancer cells following radiation (Figure 7E). While we know nothing of the pharmacokinetic properties of these compounds and cannot exclude the possibility of additional targets, these results are consistent with the notion that reducing OGG1 activity sensitizes cancer cells to radiation. Thus, both from a gene knockdown approach and a chemical inhibition approach, OGG1 contributes to the resistance of cancer cells to radiation.

## DISCUSSION

Clonogenic efficiency following ionizing radiation was decreased by CUX1 knockdown, but was increased by ectopic expression of p200 CUX1 (Figures 1 and 2). The interpretation of these results is complicated by the fact that in many tumor cell lines, CUX1 expression levels impacts on clonogenic efficiency even in the absence of radiation (Figure 1B). Further analysis suggested that in most cases the requirement for CUX1 expression in non-irradiated cells is associated with the presence of high ROS levels in tumor cells that carry a RAS mutation (Hs578T^HRAS^, MDA-MB-231^HRAS^, DLD-1^KRAS^, HCT116^KRAS^) or an oncogene that activates the RAS pathway (HT29^BRAF^ and HCC827^EGFR^) (Figure 1D). Indeed, in a previous study we demonstrated that the synthetic lethality of *CUX1* knockdown in DLD-1 cells, first reported by the group of Steve Elledge [63], was linked to its role as an auxiliary factor that stimulates the repair of oxidative DNA damage [53]. We further showed that CUX1 prevents RAS-induced senescence in primary cells by accelerating the repair of oxidative DNA lesions [53].

We noted one exception to the association between activation of the RAS pathway and high ROS: the A549 cell line, which carries a KRAS oncogene, exhibited low ROS (Figure 1D). In A549 cells, an inactivating mutation within the KEAP1 tumor suppressor gene reduces the affinity of KEAP1 for the NRF2 transcription factor, leading to greater accumulation of NRF2 in the nucleus and increased activation of genes coding for antioxidants [64].

Notwithstanding the effect of CUX1 in the absence of radiation, our results clearly established that *CUX1* expression level impacts on the response of cancer cells to radiation (Figures 1 and 2). We also presented results showing that in addition to its transcriptional function, the role of CUX1 in DNA repair also plays an important role in radioresistance. We first review previous evidence implicating the transcriptional functions of CUX1 and then discuss results from the present study supporting a direct role of CUT domains in the repair of oxidative DNA damage.

There are two main CUX1 protein isoforms: a full-length p200 isoform that functions as an auxiliary factor in base excision repair [53, 55], and a shorter p110 isoform that is generated by proteolytic processing of p200 CUX1 and functions as a transcription factor (reviewed in [40, 65, 66]. We previously established that p110 CUX1 activates expression of many genes involved in DNA damage response, including the ATM, ATR, CHK1 and CHK2 checkpoint kinases [48]. Consistently, RNAi knockdown or genetic inactivation of CUX1 reduces ATM expression and negatively impacts protective responses mediated by ATM following exposure to radiation. These results provided compelling evidence that adequate basal DNA damage response protein levels depend on CUX1 transcriptional regulation, and must be in place prior to DNA damage such that cells can rapidly respond to mutagenic insult [48]. Additionally, p110 CUX1 stimulates expression of many genes involved in the spindle assembly checkpoint, whereas CUX1 knockdown causes a decrease in the expression of these genes [47]. The latter transcriptional activity of p110 CUX1 most likely explains the deleterious effect of CUX1 knockdown on T98G cells (Figure 1B). Knockdown of BUBR1, a direct transcriptional target of p110 CUX1, provokes cell death in T98G cells [67]. This effect has been attributed to the fact that T98G cells are hyperpentaploid [68], and therefore require a robust mitotic checkpoint to ensure bipolar mitosis and the production of viable daughter cells [67, 69].

In the present study, we showed that the full-length CUX1 protein is rapidly recruited to the sites of DNA damage, suggesting a direct role in the response to DNA damage (Figures 3 and 4). We showed that a recombinant protein containing CUT domains 1 and 2, C1C2, is sufficient for recruitment to DNA damage and increased survival of DLD-1 cells submitted to ionizing radiation (Figures 4 and 5E). Ectopic expression of this CUT domain protein accelerated the cleavage at 8-oxoG in whole cell extracts, while the same assay performed *in vitro* with purified proteins demonstrated that CUT domains stimulate the glycosylase and AP/lyase activities of OGG1 (Figure 5C and 5D). Importantly, this CUT domain protein does not function as a transcriptional activator and does not stimulate expression of genes involved in base excision repair [53, 55]. Therefore, increased survival conferred by the C1C2 recombinant protein must result from its role in DNA repair.

Could the stimulation of OGG1 by CUT domains contribute to the increased resistance of cancer cells to radiation? To answer this question, we first tested the effect of OGG1 overexpression and observed that OGG1 increases the survival of DLD-1 cancer cells following radiation (Figure 5E). These results differ from previous studies showing that higher OGG1 and NTH1 expression sensitizes TK6 lymphoblastoid cells to radiation [57, 58]. Similarly, in our study ectopic expression of OGG1 did not increase resistance to radiation in RPE1 cells (Figure 5E). Clearly, OGG1 overexpression does not have the same effect, and may even have opposite effects, in different cell lines. One important difference between tested cell lines is that DLD-1 cells harbor a *KRAS* oncogene. Cells that express an activated RAS oncogene produce an excess of reactive oxygen species (ROS) that cause oxidative DNA damage [70–73]. In primary cells, this process leads to cellular senescence ([74–79]; reviewed in [80, 81]). However, one adaptive response to oxidative stress in RAS-transformed cells is the upregulation of the pathway that repairs oxidative DNA damage [53]. In support of this notion, among the synthetic lethal interactions with *KRAS* discovered in the genome-wide RNAi screen conducted by the Elledge group were 5 genes that encode proteins involved in base excision repair: NEIL2, CUX1, XRCC1, POLβ, and LIG3 [63]. In the present study, higher levels of APE1, PARP1 and POLβ were expressed in DLD-1 cells than in RPE1 cells (Figure 5G). Ectopic expression of OGG1 in cells like RPE1 and TK6 that do not express corresponding levels of proteins involved in downstream steps of base excision repair would not be expected to confer radioresistance and may even sensitize cells to radiation by increasing the number of DNA strand-breaks, as previously documented [57, 58]. In contrast, in cancer cells that have adapted to higher level of oxidative DNA damage by increasing BER gene expression, OGG1 can confer protection against damage caused by radiation, as observed in DLD-1 cells (Figure 5E).

As an alternative approach to verify the link between the stimulation of OGG1 and increased survival after ionizing radiation, we tested the effect of a CUT domain mutant, C1-2Ala, that does not stimulate OGG1 to the same extent as the wild type C1 domain (Figure 6B). In contrast to the wild type protein, the C1-2Ala mutant did not increase survival following radiation (Figure 6D and 6E).

OGG1 knockdown by three distinct siRNA sensitized DLD-1 cancer cells to radiation (Figure 7B and 7C). In agreement with these findings, treatment of cells with two compounds that inhibit OGG1 *in vitro* also sensitized DLD-1 cells to radiation (Figure 7E). These results are also consistent with those of a previous study showing that NEIL1 knockdown causes modest radiosensitization [82]. Altogether these results demonstrate that OGG1 plays an important role in reducing cytotoxic effects of radiation. Although there is considerable overlap in substrate specificities among DNA glycosylases that repair oxidative DNA lesions, OGG1 has been shown to be most important in the repair of 8-oxoG and FapyG ([83–85], reviewed in [35]). In our biochemical assays to measure OGG1 activity, we used oligonucleotides containing an 8-oxoG base because this altered base is commercially available. However, FapyG are more abundant than 8-oxoG in normal mammalian DNA [83, 86], after oxidative stress [87–89], and following treatment of cancer cells with radiation [90]. Both 8-oxoG and FapyG are mutagenic, leading to misincorporation of adenine opposite the lesion [91, 92]. In addition, FapyG is believed to be cytotoxic since it causes moderate inhibition of DNA synthesis ([24, 93], reviewed in [27]).

In summary, our results have uncovered the requirement for CUX1 expression in cancer cells with elevated ROS levels. This represents yet another case of stress phenotype of cancer cells and non-oncogene addiction, according to the concepts developed in a recent review [94]. Importantly, the present study validates CUX1 and more specifically the CUT domain as therapeutic target. Many drugs that inhibit BER enzymes, notably PARP1 and APE1, are currently tested in the clinic with various treatment modalities (https://clinicaltrials.gov/). The drawback of such approaches is that these BER enzymes perform essential functions in all tissues, since over 30,000 base alterations/day are produced in a normal human cell [95]. In contrast, *CUX1* only functions as an auxiliary factor that accelerates repair of oxidative damage [53, 55]. While *CUX1* knockdown is synthetic lethal to many cancer cells and reduces survival of all cancer cells following ionizing radiation (Figure 1), *CUX1* is not essential in normal cells as demonstrated from lethality screens in human cells [96], , the lack of effect of *CUX1* knockdown in the DLD-1 derivative cell line, DKO-4, in which the KRAS oncogene has been removed [53], and the viability of *Cux1^−/−^* knockout mice [97–99]. In addition, *CUX1* gene copy number is increased in over 70% of human cancers, and its expression inversely correlates with patient survival ([50, 100, 101]; reviewed in [40]). Together these features suggest that the CUT domain would represent an ideal therapeutic target.

## MATERIALS AND METHODS

### Cell culture

Human cell lines, (breast carcinomas Hs578T and MDA-MB-231; colorectal cancer HT29, DLD-1, and HCT116; glioblastoma U251 and T98G; lung carcinoma HCC827 and A549; hTERT-immortalized RPE1) were cultured following provided instructions. The cells were maintained in either Dulbecco's modified Eagle medium (DMEM)-high glucose, DMEM-F12, McCoy or RPMI-1640 (Wisent), supplemented with 10% Fetal Bovine Serum (Tetracycline-free; Gibco), penicillin–streptomycin (Invitrogen), and maintained at 37°C, 5% CO_2_ and atmospheric O_2_.

### Plasmid constructions

The pLXSN-p200 CUX1 retroviral construct expressing amino acids 1-1505, with a Myc tag at the N-terminus and hemagglutinin (HA) tag at the C-terminus has previously been described [49]. CUX1 C1C2-NLS lentiviral constructs were generated by inserting CUX1 fragment 522-1027 tagged with a nuclear localization signal into pLenti6 (Thermo Fisher Scientific). pLXSN-p200 CUX1-GFP and pLenti C1C2-NLS-HA-GFP were produced by inserting the EGFP sequence into the respective vectors. His-C1C2 constructs for bacterial expression were expressed in pET30a. Wild type C1 and C1 with two point mutations replacing glutamic acids with alanine (C1-2Ala) at positions 555 and 562 were synthesized as gBlocks gene fragments (Integrated DNA Technologies); for expression in mammalian cells, a nuclear localization signal and a HA tag were added at the 3′end, with flanking attB sequences for transfer into pLenti expression vector; for expression in bacteria, a His tag was added at the 5′end, with flanking attB sequences for transfer into pDest14 (Invitrogen) according to manufacturer's instructions pEGFP-hOGG1 used in live cell imaging was a generous gift from Dr. Pablo Radicella [102].

### Generation of stable cell lines

Retroviruses were produced using 293VSV cells that were co-transfected with pLXSN-p200 CUX1-HA with packaging plasmids pVPack-GP and pVPack-VSV-G (Stratagene). Lentiviruses were produced by co-transfecting 293-FT cells with plasmids pLenti humanOGG1 (ThermoScientific), pLenti-C1C2-NLS-HA, pLenti C1-NLS-HA, pLenti C1-2Ala-NLS-HA, pTRIPZ-DoxOn-shCUX1 plasmid (OpenBiosystems), packaging plasmid psPAX2 and envelop plasmid pMD2G [53]. The medium of the transfected cells containing the retrovirus and lentivirus were collected for 5 and 3 days respectively, starting 48 hours post-transfection. Viruses were applied to cells along with 6 μg/ml polybrene and cells were centrifuged at 1200 *g* for 1 h. Infected cells were selected and maintained with specific antibiotics, blasticidin, G418 or puromycin. Expression of CUX1-shRNA was induced in the stably infected pTRIPZ-DoxOn-shCUX1 cells by supplementing the growth media with 1 μg/ml of doxycycline. Cells grown in the absence of doxycycline were used as a control. Over-expression of different genes or knockdown of CUX1 was confirmed by immunoblot analysis.

### Immunoblotting and measurement of mRNA

Protein extraction and immunoblotting were conducted as described [53]. The following antibodies were used: anti-CUX1 861 (1:1000) [42], anti-HA.11 (1:1000, Covance, MMS-101R), anti-OGG1 (1:1000; Pierce, PA1-31402), and anti-tubulin (1:1000; Sigma, T6557). RNA was extracted using RNeasy Mini Kit (Qiagen), and cDNA was prepared using QuantiTect reverse transcriptase kit (Qiagen) following the manufacturer's instructions. Real time PCR was performed on Mastercycler (Eppendorf) using specific primer pairs for each gene (Supplementary Table 1).

### siRNA knockdown

OGG1 knockdown was performed by transfecting cells with three siRNA dicer constructs specific for human OGG1 mRNA (hs.Ri.OGG1.13.1, hs.Ri.OGG1.13.2 and hs.RiOGG1.13.4 (Integrated DNA Technologies) using Lipofectamine3000 (Invitrogen) according to the manufacturer's instructions. Knockdown was performed 3 days prior to radiation, and clonogenic survival assays. Expression levels were confirmed by RT-PCR and immunoblot.

### Bacterial protein purification

Expression of his-tagged fusion proteins containing C1C2, C1 (wild type) or C1 2ALA (2 alanine; mutant) was induced with isopropyl-β-D-thiogalactopyranoside in the BL21 strain of *Escherichia coli* as previously described [55]. Upon purification using nickel bound beads, several buffer exchanges were carried in 3.5-kDa molecular weight cut-off dialysis membrane (Spectra/Por 3; SpectrumLabs) to bring down imidazole concentration to less than 0.1 μM.

### Clonogenic survival assay

Clonogenic ability of irradiated cells was conducted as described previously [48]. Briefly, cells were exposed to irradiation at doses 1, 2, 4, and 5 Gy using an X-ray source biological irradiator (Rad-Source RS2000). 100–250 cells were then plated in either 60 mm or 6-well plates in triplicate. Different cell densities were plated to ensure that sufficient cell colonies were observed in all conditions. After 10–14 days of incubation, cells were washed with phosphate-buffered saline (PBS), fixed with cold methanol for 20 min then stained with 0.1% crystal violet (Acros Organics) in 20% methanol for 30 min. The number of colonies with 50 cells or more was counted. Clonogenic efficiency is represented as the percentage of seeded cells that gave rise to clones under control conditions either empty vector cells with no irradiation or untreated cells. The reported values are the averages ± standard deviations.

### Reactive oxygen species (ROS) measurements

The intracellular levels of ROS were measured by flow cytometry of live cells stained with the oxidant sensitive probe 5-(and-6)-chloromethyl-2′, 7′-dichlorodihydrofluorescein diacetate, acetyl ester (CM-H_2_DCFDA, Thermo Fisher Scientific), as recommended by manufacturer's instructions. FACS analyses were carried out on a FACScalibur machine using the CellQuestPro software. Geometric means of the fluorescence intensity of each cell line were calculated using FlowJo 887 software. The fluorescence intensity of each cell line was normalized relative to the background fluorescence value before dye was added. All measurements were done in triplicates.

### Methyl-^14^C thymidine incorporation

DLD-1 cells exposed to vehicle (DMSO) or 10 μM of chemical molecules (Chembridge 5245457 and 5552704) for 2 h prior to irradiation (2 Gy). Cells were then plated at a density of 4 × 10^3^ cells per well in 96-well Cytostar-T scintillating microplates (PerkinElmer) in the presence of vehicle or 10 μM chemical molecules. Cells were incubated in 100 μl of media with 0.5 μCi/ml of ^14^C thymidine. The incorporated thymidine was quantified twice a day with a microplate counter (MicroBeta2, PerkinElmer). Each time point was done in triplicate, and the averages ± standard deviations were calculated.

### *In vitro* 8-oxoG fluorogenic cleavage assay

Cleavage reactions with bacterially purified proteins were conducted using 50 nM hOGG1 (New England Biolabs), and 200 nM of BSA, His-C1C2, His-C1 or His-C1-2Ala in 50 mM Tris (pH 7.1), 1 mM EDTA, and 20 mM KCl. In Figure 7, cleavage assay was performed in the presence of either vehicle (DMSO) or 10 μM of chemical molecules (Chembridge 5245457 and 5552704). Reactions with total cell extracts were performed as described by [103], with slight modification. Briefly, 60 μg of total proteins were used with 100 ng of poly(dI-dC) as a nonspecific competitor DNA. In both cases, 40 nM of molecular beacon comprised of deoxyoligonucleotide containing a 8-oxoG lesion (IDT) was used as previously described [104]. Briefly, a molecular beacon is 43 bases with 6-Carboxyfluorescein (6-FAM) moiety conjugated to the 5′end and a Dabcyl moiety conjugated to the 3′ end of the oligonucleotide. The sequence is designed to create a stem-loop structure with 13 nucleotide loop and 15 base pair stem where the 6-FAM fluorescence is efficiently quenched by Dabcyl. The 8-oxoG is placed on the 6^th^ nucleotide of the stem. The reactions were incubated at 37°C, and fluorescence data were collected on Mastercycler (Eppendorf) equipped with standard optics (excitation filter, 465 nm; emission filter, 510 nm). Each reaction was done in triplicate, and the averages ± standard deviations were calculated.

### Laser-induced DNA damage, live-cell imaging and quantitation (GFP-CUX1)

Laser microirradiation-induced DNA damage was produced by three methods. In the first method, cells were plated onto a 4 well Lab-Tek chambered coverglass (NUNC 155383) or chamber slides (NUNC 177399) and imaged with or without transfection the following day with the desired GFP fusion plasmid [105]. 4–6 hours post-transfection media was replaced with DMEM media supplemented with 10 μM 5-Iodo-2′-deoxyuridine to favor the production of double-strand breaks by UV-laser radiation. (IdU, Sigma I7125). Next day live-cell imaging experiment was performed using the Zeiss LSM 510 Meta laser scanning confocal microscope. Cells were visualized using 63x water immersion objective. DNA within a narrow rectangular region in the nucleus was damaged using 500 iterations of the fast line scan with UV (351/364 nm) laser operated at 75% of maximum output. Images were acquired immediately before DNA damage, immediately after DNA damage and periodically thereafter using the Argon laser (488 nm). For immunocytochemical staining, laser treatment was performed the following day using an Axiovert 200 M integrated with PALM microlaser workstation equipped with a 337 nm UV laser. Narrow linear regions within nuclei were marked for UV laser irradiation using PALM robo v3.2 software. UVA irradiation (30 Hz, 337 nm) was delivered in the demarcated regions using a 40× objective. Cells were either fixed immediately or returned to the incubator and fixed at the desired time point followed by immunocytochemical staining, as described below. In the third method, cells were plated in 30 mm glass bottom dishes (Matek) and 24 h later were transfected with GFP fusion plasmids as indicated. The following day, cells were subjected to 405-nm laser irradiation, as previously described [106]. Briefly, cells pretreated with 2 μM Hoechst 33342 (Sigma-Aldrich) for 5 min before being imaged at 37°C using a custom-built microscope (Cell Observer; Carl Zeiss/Intelligent Imaging Innovations), equipped with a heated CO_2_ incubator, diode-based lasers (405, 488, 561, and 633 nm), and a spinning-disk confocal scanning unit (CSU-X1; Yokogawa Electric Corporation) using a 40×, 1.4 NA immersion oil objective lens. UV laser damage was induced by a 100 mW, 405 nm diode laser using a Vector Scan Unit (Intelligent Imaging Innovations), where the effective light output was measured as ~8 mW at the objective when using 100% power. A single line scan of the 405 nm laser at 70% power was sufficient to generate DNA DSBs, which was estimated to be equivalent to ~40–60 Gy cellular dose. Images were captured every 5 s for 5 min using an electron-multiplying charge-coupled device camera (Evolve; Photometrics) and SlideBook 5.5 software (Intelligent Imaging Innovations).

### Immunocytochemistry

Cells were washed with PBS and fixed with 4% paraformaldehyde (PFA) for 10 min at room temperature. Cells were permeabilized using 0.5% Triton X-100 (10 min, room temperature), washed with PBS and then incubated with primary antibodies in 5% normal goat serum (Vector Laboratories H-1200). After washing cells were incubated with secondary antibodies in 5% normal goat serum and washed again. Finally, the cells were mounted using mounting media containing DAPI (Vector Laboratories S-1000). All solutions were made in PBS. Slides were visualized and images captured using either Nikon Eclipse 80i or Zeiss Axiovert 200 M fluorescence microscopes.

## SUPPLEMENTARY MATERIALS TABLE AND MOVIES












