# Supplementary material for: The DNA repair function of CUX1 contributes to radioresistance
Source: Oncotarget. 2017 Jan 28;8(12):19021–38. doi: 10.18632/oncotarget.14875 (PMC5386666; doi:10.18632/oncotarget.14875)
Supplement: Supplementary file 1 [file oncotarget-08-19021-s001.pdf]

The DNA repair function of *CUX1* contributes to radioresistance

Supplementary Materials

Supplementary Table 1: Primer sequences used for PCR amplification

	Primers for PCR amplification
APE1	(F) GATTGGGTAAAGGAAGAAGC<br>(R) TCAAATTCAGCCACAATCAC
OGG1	(F) GTTCCTCCAACAACAACAT<br>(R) AGATGCAGTCAGCCACCTTGG
PARP1	(F) CAACTTTGCTGGGATCCTGT<br>(R) TGTTTCCAAGGGCAACTTCT
POLβ	(F) AGACTCTCAACGGGGGAATCAC<br>(R) GCAGATGGACCAATGCCACTAAC
XRCC1	(F) CAGACCAAAACCAAGCCCACTC<br>(R) TCACTGTCCGTGTTCTCATCCG
GAPDH	(F) ACCACAGTCCATGGCATCAC<br>(R) TCCACCACCCTGTTGCTGTA

Supplementary Movie 1: GFP-p200 CUX1 is rapidly recruited to sites of DNA damage upon 351/364 nm laser microirradiation. See Supplementary_Movie_1

Supplementary Movie 2: p200 CUX1-GFP is rapidly recruited to sites os DNA damage upon 405 nm laser microirradiation. See Supplementary_Movie_2

Supplementary Movie 3: Cut domains are rapidly recruited to sites of DNA damage upon 405 nm laser microirradiation. See Supplementary_Movie_3

Supplementary Movie 4: OGG1-GFP is recruited to sites of DNA damage upon 405 nm laser microirradiation. See Supplementary_Movie_4

Supplementary Movie 5: Ku-mcherry is recruited to sites of DNA damage upon 405 nm laser microirradiation. See Supplementary_Movie_5
